# Measurement of ^131^I activity in air indoor Polish nuclear medical hospital as a tool for an internal dose assessment

**DOI:** 10.1007/s00411-017-0724-3

**Published:** 2017-12-14

**Authors:** K. Brudecki, A. Szczodry, T. Mróz, A. Kowalska, J. W. Mietelski

**Affiliations:** 1 Institute of Nuclear Physics, Polish Academy of Sciences, Radzikowskiego 152, 31-342 Krakow, Poland; 2 Department of Endocrinology and Nuclear Medicine Holycross Cancer Center, Artwińskiego 3, 25-734 Kielce, Poland; 3 Pedagogical University in Cracow, Podchorążych 2, 30-084 Krakow, Poland

**Keywords:** ^131^I, Air, Nuclear medicine, Medical personnel, Equivalent and effective doses

## Abstract

This paper presents results of ^131^I air activity measurements performed within nuclear medical hospitals as a tool for internal dose assessment. The study was conducted at a place of preparation and administration of ^131^I (“hot room”) and at a nurse station. ^131^I activity measurements were performed for 5 and 4 consecutive working days, at the “hot room” and nurse station, respectively. Iodine from the air was collected by a mobile HVS-30 aerosol sampler combined with a gas sampler. Both the gaseous and aerosol fractions were measurement. The activities in the gaseous fraction ranged from (28 ± 1 Bq m^−3^) to (492 ± 4) Bq m^−3^. At both sampling sites, the activity of the gaseous iodine fraction trapped on activated charcoal was significantly higher than that of the aerosol fraction captured on Petrianov filter cloth. Based on these results, an attempt has been made to estimate annual inhalation effective doses, which were found to range from 0.47 mSv (nurse female) to 1.3 mSv (technician male). The highest annual inhalation equivalent doses have been found for thyroid as 32, 27, 13, and 11 mSv, respectively, for technician male, technical female, nurse male, and nurse female. The method presented here allows to fill the gaps in internal doses measurements. Moreover, because method has been successful used for many years in radioactive contamination monitoring of air in cases of serious nuclear accidents, it should also be used in nuclear medicine.

## Introduction

Among the radionuclides used in medical diagnostics and therapy, ^131^I is very popular; because it is a beta emitter, it has a short half-life (*T*_1/2_ = 8.03 d) and it is selectively taken up by the thyroid. In the previous studies, we focused on measuring the activity of ^131^I in thyroids of nuclear medicine staff and calculating internal doses. For example, in 2016, the ^131^I thyroid activity was measured in 30 members of nuclear medicine personnel of the Department of Endocrinology and Nuclear Medicine Holycross Cancer Centre (E&NM HCC) in Kielce, Poland. Measurements were conducted using the Whole-Body Spectrometer (WBS) equipped with two HPGe detectors. ^131^I activity in thyroids was observed in ten individuals. The measured activities were found to vary between (5 ± 2) Bq and (217 ± 56) Bq. Estimated internal annual effective doses ranged from 0.02 to 0.8 mSv. The corresponding annual thyroid equivalent doses ranged from 0.4 to 15.5 mSv (Brudecki et al. 2017a).

The previous studies have shown that radiological protection of workers being only based on measurements with thermoluminescence detectors (TLDs) is insufficient. In such a case, exposures to the external nuclear radiation field are monitored, while those due to incorporated radionuclides are not. On the other hand, a WBS as a tool for evaluating internal doses is highly impractical, because it is an expensive and complex instrument, and its handling requires expert knowledge. At present in Poland, only two such devices are in operation, while there exist more than 60 nuclear medicine facilities using radioactive iodine. In principle, internal dose assessment can be also done by measuring ^131^I activity concentrations in the air within nuclear medical hospitals (ICRP 1997). Some results based on this approach were already published. For example, Hoi et al. (2017) reported measured average activity concentrations of ^131^I in the air of 815 ± 37 Bq m^−3^ resulting in effective doses for eight workers between 0.146 ± 0.004 and 3.052 ± 0.095 mSv. Measurements performed by Jiemwutthisak et al. (2012) in a nuclear medicine hospital showed ^131^I activity concentrations between 2.94 ± 3.60 Bq m^−3^ in a hospital ward waste collection area and 31.6 ± 16.3 Bq m^−3^ in a fume hood, where the iodine pills are taken out of the safety containers and prepared for patients. Results published by Ferdous et al. (2017) showed ^131^I activity concentrations from 0.19 to 60.67 Bq m^−3^ (uncertainties were not expressed) in the hot lab of a nuclear medicine facility.

The first step of the present study was to measure the ^131^I activity concentrations in the air of rooms used for radioiodine treatment, at the Department E&NM HCC in Kielce, Poland. A two-way monitoring system was applied in which activities of radioiodine associated with gaseous and aerosol fractions can be measured separately. As a second step, inhalation doses for medical personnel were estimated according to a methodology recommended by the International Commission on Radiological Protection (ICRP).

## Materials and methods

### Aerosol and gas sampling

Because gaseous and aerosol fractions of ^131^I activity in the air are not only from “hot rooms” (rooms prepared for handling Na^131^I pills), but may also be from patients (e.g., perspiration and breath) (Ibis et al. 1992), aerosol/gas activity was measured in samples collected from different locations within the investigated nuclear medicine facility located at the Department E&NM HCC in Kielce, Poland. ^131^I activity concentrations were measured in samples collected in a “hot room” and a nurse station. Measurements were performed for 5 and 4 consecutive working days, respectively, for the “hot room” and the nurse station.

**Fig. 1:**
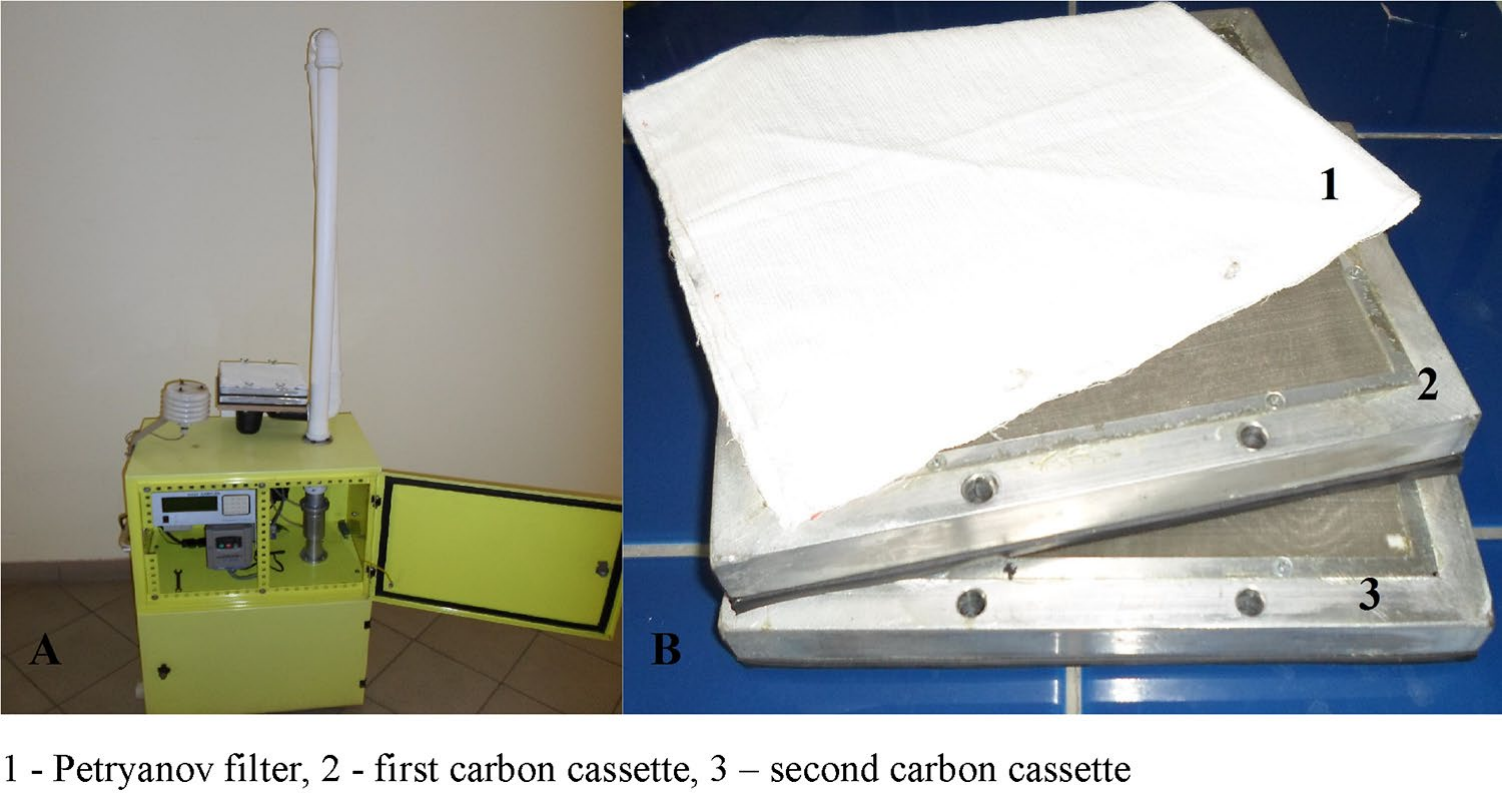
Air sampler (**a**) and filters (**b**). 1—Petryanov filter, 2—first carbon cassette, 3—second carbon cassette

For sample collection, a mobile aerosol sampler HVS-30 (produced by Atmoservice Ltd., Poznań, Poland) combined with a gas sampler was used (Mietelski et al. 2005). The HVS-30 aerosol sampler operated at a flow rate of 30 m^3^ h^−1^. The aerosol fraction was captured using a Petryanov filter FPP-15-1.5 [poly(vinyl chloride)], while the gas fraction was collected using granular-activated carbon as a sorbent (IBJ-6, mesh size 2 mm, produced by Gryskand, Hajnówka, Poland). The sampler and the filters are shown in Fig. 1. The carbon used was impregnated with KI which means that both organic and inorganic iodines were collected (Wilhelm 1982; Wangchang et al. 1993). Such a method has already been successfully applied for measurement of aerosol and gas activities (Mietelski et al. 2005, 2014, 2017; Masson et al. 2011). Before sampling, the charcoal was heated to 105 °C for 2 days to remove moisture. Then, the charcoal was sealed in plastic bags. After sampling, the charcoal was again placed in plastic bags and transported to the laboratory for gamma-ray spectrometry.

The charcoal was localized in two identical cassettes forming a single cartridge. The air was pumped through the cartridge, subsequently through the first and second cassettes. The same absorption yield is assumed for both cassettes; therefore, it can be assumed that the activity concentration in the cassettes forms geometrical series. Thus, the total activity concentration in the air ${C_{total}}$ can be calculated from the known formula (Eq. 1) as a sum of an infinite geometric series:

1 $${C_{{\text{total}}}}=\frac{{{C_A}}}{{1 - q}}$$

where $q=\frac{{{C_A}}}{{{C_B}}}$, and ${C_A}$ and ${C_B}$ represent the activity concentrations measured in the first and second cassettes, respectively.

In such a case, the total efficiency $\varepsilon$ of absorption can be calculated, as shown in

2 $$\varepsilon =1 - {q^2}.$$

To make sure that the samples can be considered representative, separated areas with normally functioning ventilation and air conditioning were chosen as sampling locations. Following recommendations of the International Atomic Energy Agency (IAEA), the sampling cartridge was mounted 1.6 m above the floor to collect the air from the breathing zone (IAEA 1999).

### Gamma spectrometric analysis

For determination of ^131^I activity collected on charcoal and Petryanov filter cloth, the intensity of 364.46 kev gamma line was measured by means of a low-background gamma spectrometer including an HPGe detector. Before the Petryanov filters could be measured, they had to be compressed into a disc-shaped geometry of 5 cm diameter and about 4 mm height. The charcoal from each cassette was measured inside 0.5 L plastic Marinelli beakers. The efficiency calibration was determined with a multi-gamma source (SZN 40/10 provided by Polatom) and a standard mixed gamma source (SZM-3 provided by Polatom) for the filters and charcoal samples, respectively.

### Doses estimation

Based on the measured ^131^I air activity concentrations, doses were estimated for the technicians and nurses, separately for the gas and aerosol fraction.

From the routine schemes of the medical staff and from survey questions, working times in the contaminated areas and breathing parameters were estimated. Normally, nurses work one 12-h shift every 3 days in the department, for 3 months in the year. Furthermore, ^131^I is administered to patients weekly on Fridays, so typically, the technicians spent between 4 and 8 h (depending on the patient number) per week in the “hot room”. Here, for the dose calculations, an average value of 6 h is assumed. In the present study, the human respiratory tract model published in ICRP (1994) was used. The breathing parameters assumed for the dose computations are presented in Table 1.

**Table 1:** Time budget, ventilation parameters, and activity concentrations assumed for the dose calculations

Parameters\profession	Technician	Nurse
Exercise level	Sitting	Sitting
Breathing rate (m^3^ h^−1^)	0.39 (females), 0.54 (males)	
Single intake time (h)	6	12
Single ^131^I gas fraction activity in the air (Bq m^−3^)	490	170
Single ^131^I aerosol fraction activity in the air (Bq m^−3^)	7	18
Intake frequency	Once a week (on Friday)	Every 3 days
Number of intakes per year	50	30

Unfortunately, the measuring equipment used in the present study did not allow measurement of aerosol diameters. Therefore, the deposition of ^131^I aerosol fractions was calculated for an assumed activity median aerodynamic diameter (AMADs) of the attached aerosols of 5 µm, following the ICRP recommendation for workers. Deposition of the gas fraction was calculated for elemental iodine (100% deposition in the respiratory tract, 10% in the extrathoracic region—ET_1_, 40% in posterior nasal passages—ET_2_, and 50% in the bronchial region—BB) (ICRP 1995). Both simplifications resulted in an overestimation of the calculated doses which is, however, in line with the so-called conservative assessment rule, where doses should be over-rather than underestimated. Details on deposition parameters are presented in Table 2.

**Table 2:** Deposition parameters used in the present study (ICRP 1995, 2002)

Region	5 µm AMAD and sitting subjects		Gas	
	Adult male	Adult female	Adult male	Adult female
ET_1_	2.80 × 10^− 1^	2.70 × 10^− 1^	1.00 × 10^− 1^	
ET_2_	3.40 × 10^− 1^	3.30 × 10^− 1^	4.00 × 10^− 1^	
BB _fast and seq_	1.20 × 10^− 2^	1.10 × 10^− 2^	5.00 × 10^− 1^	
BB _slow_	4.30 × 10^− 3^	3.90 × 10^− 3^		
bb _fast and seq_	1.50 × 10^− 2^	1.80 × 10^− 2^	0	
bb _slow_	8.70 × 10^− 3^	1.00 × 10^− 2^		
AI	1.00 × 10^− 1^	9.40 × 10^− 2^	0	

*AMAD* activity median aerodynamic diameter, *ET*_*1*_ extrathoracic region, *ET*_*2*_ posterior nasal passages, *BB* bronchial region, *bb* bronchiolar region, *AI* alveolar–interstitial region

Doses were calculated based on the time-integrated ^131^I activity, radiation-weighted coefficients (*S*_w_), and tissue-weighting factors (*w*_T_) according to the ICRP methodology. The time-integrated ^131^I activity was obtained from computer biokinetic modeling. Here, the iodine biokinetic model developed by Leggett (2010) was combined with the human respiratory tract model (ICRP 1994, 2002) and gastro-intestinal tract model (ICRP 1979) developed by ICRP. The SAAM II software (Epsilon Group, VA, USA) was used for the calculations. This overall methodology has already been successfully applied previously (Brudecki et al. 2014, 2017a, b; Li et al. 2008). The radiation-weighted *S*_w_ coefficients were calculated separately for males and females, with the SEECAL program (Oak Ridge National Laboratory, Oak Ridge, TN, USA). Tissue-weighting factors, w_T_, were taken from ICRP 103 (2007).

## Results and discussion

At both sampling sites, the activity of the gaseous iodine fraction trapped on activated charcoal was significantly higher than that of the aerosol fraction captured on Petrianov filter cloth. The highest gaseous activity concentration was measured on 17 October 2016 in the air of the “hot room” (492 ± 4 Bq m^−3^). On that day, tablets with radioactive iodine were delivered and prepared for patients. The lowest gaseous activity concentration was measured on 23 October 2016 (28 ± 1 Bq m^−3^), 1 day before the next delivery of radioiodine tablets. The aerosol activity concentration in the air of the “hot room” was also slightly decreasing in the course of this week (from 7.2 ± 0.6 to 5.2 ± 0.3 Bq m^−3^). Gaseous activity concentrations measured in the air of the nurse station were on a similar level during the whole study period, and ranged from 146 ± 2 to 208 ± 3 Bq m^−3^ with a mean value of 174 ± 25 Bq m^−3^. Because the nurse station is located on the corridor next to the patients’ rooms and because there is no correlation between iodine delivery and activity variations, the measured mean activity concentration probably represents the average activity concentration of ^131^I in the air of corridor and patients’ rooms. For details, see Table 3.

**Table 3:** Measured ^131^I air activity concentrations in the air; given uncertainties represent measurement error calculated from one sigma counting statistics

Place	Date	^131^I activity concentration, gas fraction (Bq m^−3^)	^131^I activity concentration, aerosol fraction (Bq m^−3^)
“Hot room”	17 October 2016	492 ± 4	7.2 ± 0.6
	20 October 2016	280 ± 3	6.2 ± 0.6
	21 October 2016	42 ± 1	6.1 ± 0.5
	22 October 2016	34 ± 1	5.2 ± 0.3
	23 October 2016	28 ± 1	5.7 ± 0.4
Nurse station	18 November 2016	171 ± 2	18 ± 1
	21 November 2016	173 ± 3	40 ± 1
	22 November 2016	208 ± 3	8.5 ± 0.9
	23 November 2016	146 ± 2	6.9 ± 0.9
	Average	174 ± 25	18 ± 15

On the basis of the results obtained for ^131^I air activity concentrations, radiation doses from inhalation of ^131^I were estimated. It turned out that the doses received from inhalation of the ^131^I aerosol fraction were significant smaller than those received from the ^131^I gas fraction, and can be neglected. For the gaseous fraction, estimated effective annual doses received by the medical staff varied between 0.47 mSv (nurse, female) and 1.3 mSv (technician, male). In general, doses for females were lower than those for males. The resulting effective and equivalent doses are shown in Table 4.

**Table 4:** Calculated annual organ equivalent and effective doses (given in Sv)

	Thyroid	Effective dose
Gas fraction		
Technicans		
Male	3.2 × 10^−2^	1.3 × 10^−3^
Female	2.7 × 10^−2^	1.1 × 10^−3^
Average	3.0 × 10^−2^	1.2 × 10^−3^
Nurses		
Male	1.3 × 10^−2^	5.4 × 10^−4^
Female	1.1 × 10^−2^	4.7 × 10^−4^
Average	1.2 × 10^−2^	5.0 × 10^−4^
Aerosol fraction		
Technicans		
Male	2.5 × 10^−4^	1.0 × 10^−5^
Female	2.0 × 10^−4^	8.0 × 10^−6^
Average	2.2 × 10^−4^	9.0 × 10^−6^
Nurses		
Male	7.8 × 10^−4^	3.2 × 10^−5^
Female	6.0 × 10^−4^	2.4 × 10^−5^
Average	6.9 × 10^−4^	2.8 × 10^−5^

The highest doses were calculated for technicians working in the “hot room”. The effective annual doses for this group reached 1.1 and 1.3 mSv, respectively, for females and males. In contrast, for nurses, annual effective doses were about two times smaller (0.47 mSv for females and 0.54 mSv for males), as one might expect that the highest annual equivalent organ doses were found for the thyroid. Equivalent thyroid doses calculated for male technicians, female technicians, male nurses, and female nurses were 32, 27, 13, and 11 mSv, respectively.

Calculated effective doses are much lower than the corresponding dose limits included in Polish and European law regulations, which are limiting effective dose from occupational exposure to 20 mSv per year. They are also lower than the effective dose from natural background radiation in Poland which is 2.48 mSv per year (similar to the worldwide average exposure 2.4 mSv per year) (Janik and Tokonami 2009). However, the equivalent dose for the thyroid due to the Chernobyl accident estimated for a reference Polish inhabitant was equal to 45 mSv (Pietrzak-Flis et al. 2003), which means that a technician administering iodine accumulates over his professional career (30 years) a dose which is about 20 times higher than the dose from the Chernobyl accident.

The thyroid activities calculated in the present work based on the ^131^I air activity concentration and a biokinetic model were also compared to those measured in a previous study (Brudecki et al. 2017a). The results of this comparison are shown in Table 5. The best match was obtained for technicians, for whom the thyroid activity calculated in the present study is three-to-four times higher than that measured by Brudecki et al. 2017a. In contrast, for nurses, the calculated activity is about one order of magnitude larger than that measured. Both thyroid and air measurements were done during the normal work of the whole department. However, in the present study, air measurements were done only during 1 week for each workplace, and ^131^I activity concentrations in the air may change substantially depending on the number of patients treated. Moreover, a single exposure time of 6 h was assumed in the present work for technicians and 12 h for nurses, which represent average work times; this assumption may also contribute to the uncertainties of the doses calculated here. To reduce these uncertainties, exposure times should be monitored individually, for example, using motion detectors (Hoi et al. 2016). In addition, technicians work under similar conditions throughout the year, while nurses in the department work 3 months a year, and then they are rotated to other departments, which can influence the dose estimates.

**Table 5:** Comparison between measured and calculated thyroid activities

Subjects	Simulated thyroid activity (Bq)	Measured thyroid activity (Brudecki et al. 2017a) (Bq)
Nurses		
Male	1050	–
Female	750	from < 5 to 66 ± 17
Technicians		
Male	600	217 ± 56
Female	450	107 ± 28

## Conclusion

Up to now, internal doses were not considered important in Polish nuclear medicine facilities. Consequently, workers are only monitored for exposure to external radiation fields using TLD dosimeters. These measurements give no information about the radiation dose from incorporated radioiodine. The results obtained in the present work suggest that periodic and systematic checks and measurements of internal contamination should be an integral part of the radiation protection of staff working with high activities of ^131^I.

The method described here to monitor radiation doses from incorporated radioiodine has some drawbacks. For example, the proposed method needs a sampling station including an HPGe detector which is expensive, and it will be difficult to perform constant monitoring by hospital personnel. In addition, averaging of exposure time may represent a significant source of uncertainties in dose estimation, which should be improved by individual time monitoring.

However, despite these disadvantages, the presented method can successfully be used to monitor internal doses from incorporated radioiodine. The method has a lot of advantages. For example, collection time is relatively short (minutes or hours) which means that in a short period of time, it is possible to collect many samples from different locations. Furthermore, measurements can be carried out onsite, which does not influence nuclear medicine facility staff in fulfilling their duties. Moreover, this method has already been successful used for many years to monitor radioactive contamination in the air at places of serious nuclear accidents. It is concluded that there are no reasons not to use it also in nuclear medicine. In practice, instead of computer modeling, ICRP dose conversion factors can be used. This will significantly simplify and speed up the dose calculations.

## References

[CR1] Brudecki K, Li WB, Meisenberg O, Tschiersch J, Hoeschen C, Oeh U (2014). Age-dependent inhalation doses to members of the public from indoor short-lived radon progeny. Radiat Environ Biophys.

[CR2] Brudecki K, Kowalska A, Zagrodzki P, Szczodry A, Mroz T, Janowski P, Mietelski JW (2017). Measurement of ^131^I activity in thyroid of nuclear medical staff and internal dose assessment in a Polish nuclear medical hospital. Radiat Environ Biophys.

[CR3] Brudecki K, Szufa K, Mietelski JW (2017). ^131^I age-dependent inhalation dose in Southern Poland from Fukushima accident. Radiat Environ Biophys.

[CR5] Ferdous J, Shramin N, Begum A, Begum A (2017). Airborne radioactivity in hot lab of nuclear medicine. J Sci Res.

[CR6] Hoi TX, Phuong HT, Hung NV (2016). Using smartphone as a motion detector to collect time-microenvironment data for estimating the inhalation dose. Appl Radiat Isot.

[CR7] Hoi TX, Phuong HT, Hung NV (2017). Estimating the internal dose for ^131^I production workers from air sampling method. Radiat Prot Dosim.

[CR8] IAEA (1999). International Atomic Energy Agency. Assessment of occupational exposure due to intakes of radionuclides.

[CR9] Ibis E, Wilson CR, Collier BD, Akansel G, Istiman AT, Yoss RG (1992). Iodine-131 contamination from thyroid cancer patients. J Nucl Med.

[CR10] ICRP (1979) International Commission of Radiological Protection. Limits for intakes of radionuclides by workers. Part 1. ICRP Publication 30. Ann ICRP 2(3–4). Pergamon, Oxford 7458091

[CR11] ICRP (1994) International Commission of Radiological Protection. The human respiratorytract model for radiological protection. ICRP Publication 66. Ann ICRP 24(1–3). Pergamon, Oxford

[CR12] ICRP (1995) International Commission on Radiological Protection. Age-dependent doses to members of the public from intake of radionuclides: Part 4. Inhalation dose coefficients. ICRP Publication 71. Ann ICRP 25(3–4). Pergamon, Oxford 8735008

[CR13] ICRP (1997) International Commission on Radiological Protection. Individual monitoring for internal exposure of workers (preface and glossary missing). ICRP Publication 78. Ann ICRP 27(3–4). Pergamon, Oxford

[CR14] ICRP (2002) International Commission of Radiological Protection. Guide for the practical application of the ICRP human respiratory tract model. ICRP Supporting Guidance, vol 3. Pergamon, Oxford

[CR15] ICRP (2007). International Commission of Radiological Protection. The 2007 recommendations of the International Commission on Radiological Protection. ICRP Publication 103. Ann ICRP.

[CR16] Janik M, Tokonami S (2009). Natural and artificial sources of radioactivity in Poland. Jpn J Health Phys.

[CR17] Jiemwutthisak P, Sritongkul N, Chaudakshetrin P, Kanchanaphiboon P, Tuntawiroon M (2012). Air monitoring to control the intake of airborne radioiodine-131 contaminants by nuclear medicine workers.

[CR18] Leggett RW (2010). A physiological systems model for iodine for use in radiation protection. Radiat Res.

[CR19] Li WB, Tschiersch J, Oeh U, Hoeschen C (2008). Lung dosimetry of inhaled thoron decay products.

[CR20] Masson O, Baeza A, Bieringer J, Brudecki K, Bucci S, Cappai M, Carvalho FP, Connan O, Cosma C, Dalheimer A, Didier D, Depuydt G, De Geer LE, De Vismes A, Gini L, Groppi F, Gudnason K, Gurriaran R, Hainz D, Halldórsson O, Hammond D, Hanley O, Holeý K, Homoki Z, Ioannidou A, Isajenko K, Jankovic M, Katzlberger C, Kettunen M, Kierepko R, Kontro R, Kwakman PJM, Lecomte M, Leon Vintro L, Leppänen AP, Lind B, Lujaniene G, Mc Ginnity P, Mc Mahon C, Malá H, Manenti S, Manolopoulou M, Mattila A, Mauring A, Mietelski JW, Møller B, Nielsen SP, Nikolic J, Overwater RMW, Pálsson SE, Papastefanou C, Penev I, Pham MK, Povinec PP, Ramebäck H, Reis MC, Ringer W, Rodriguez A, Rulík P, Saey PRJ, Samsonov V, Schlosser C, Sgorbati G, Silobritiene BV, Söderström C, Sogni R, Solier L, Sonck M, Steinhauser G, Steinkopff T, Steinmann P, Stoulos S, Sýkora I, Todorovic D, Tooloutalaie N, Tositti L, Tschiersch J, Ugron A, Vagena E, Vargas A, Wershofen H, Zhukova O (2011). Tracking of airborne radionuclides from the damaged Fukushima Dai-ichi nuclear reactors by European networks. Environ Sci Technol.

[CR21] Mietelski JW, Grabowska S, Nowak T, Bogacz J, Gaca P, Bartyzel M, Budzanowski M (2005). Inhalation dose due to presence of ^131^I in air above septic tank system of an endocrinology hospital. Radiat Prot Dosim.

[CR22] Mietelski JW, Kierepko R, Brudecki K, Janowski P, Kleszcz K, Tomankiewicz E (2014). Long-range transport of gaseous 131I and other radionuclides from Fukushima accident to Southern Poland. Atmos Environ.

[CR23] Mietelski JW, Nalichowska E, Tomankiewicz E, Brudecki K, Janowski P, Kierepko R (2017). Gamma emitters in atmospheric precipitation in Krakow (Southern Poland) during the years 2005–2015. J Environ Radioact.

[CR24] Pietrzak-Flis Z, Krajewski P, Radwan I, Muramatsu Y (2003). Retrospective evaluation of 131I deposition density and thyroid dose in Poland after the Chernobyl accident. Health Phys.

[CR25] Wangchang L, Yuying H, Yianwei W, Ming J, Liangtian G (1993). Research on removal of radioiodine by charcoal.

[CR26] Wilhelm JG (1982). Iodine filters in nuclear installations.

